# Faith-based leaders’ perceptions on the implementation of programs to promote healthy lifestyles in churches in Barbados- a mixed-methods analysis

**DOI:** 10.1186/s12889-025-23245-7

**Published:** 2025-07-02

**Authors:** Natasha P. Sobers, Madhuvanti Murphy, Saria Hassan, Katrina Norville, Lisa Brathwaite-Graham, Ian Hambleton, Simon G. Anderson, Kia Lewis, Trevor S. Ferguson

**Affiliations:** 1 George Alleyne Chronic Disease Centre, Caribbean Institute for Health Research, The University of the West Indies, Cave Hill Campus, Barbados; 2 Department of Medicine, Emory University School of Medicine, Atlanta, GA USA; 3 New Testament Church of God, St. Michael, Barbados; 4 Epidemiology Research Unit, Caribbean Institute for Health Research, The University of the West Indies, Mona Campus, Jamaica

**Keywords:** Acceptability, Appropriateness, Small Island developing states, Faith-based organizations, Chronic disease self-management

## Abstract

**Background:**

There is a high burden of chronic diseases such as hypertension and diabetes in small island developing states (SIDS). SIDS governments have committed to a range of public health measures to reduce this burden including community-based health education in collaboration with civil society organizations. We sought to explore church leaders’ perceived acceptability, appropriateness, and feasibility of implementing self-management health programs in 20 faith-based organizations in the small island developing state of Barbados.

**Methods:**

This was a concurrent nested mixed methods study - a quantitative online survey and a qualitative inquiry using semi-structured interviews. Acceptability, appropriateness and feasibility of the intervention were assessed using the following quantitative assessment tools: Acceptability of Intervention Measure (AIM), Intervention Appropriateness Measure (IAM) and Feasibility of Intervention Measure (FIM). Thirteen semi-structured interviews were conducted virtually, recorded and transcribed verbatim. Transcripts were analyzed using thematic analysis based on deductive codes from Proctor’s implementation outcomes definitions.

**Results:**

From the 52 respondents of the survey, the median and interquartile ranges for the AIM, IAM and FIM scales were 16 (15-20), 16 (16-20) and 16 (15-17) (out of 20), respectively. We found high levels of acceptability, 82% (95% CI (69%, 95%)) of leaders indicating that health programs in churches met with their approval; and high levels of appropriateness- 90% (95% CI (80%, 100%)) indicating health programs in churches were “fitting” and “a good match”. In interviews, leaders expressed acceptance of healthy lifestyle programs and described their appropriateness through alignment with church doctrines stating, “the body is the temple of God”. Feasibility scores were low, only 60% (95% CI (44%, 76%)) indicated that health programs in churches would be easy to use. Leaders felt that the high cost of nutritious food and restricted finances exacerbated by COVID-19 were likely to be barriers to program success. They felt unprepared to address health-related topics and expressed the need for support from healthcare providers who are sensitive and respectful of church culture.

**Conclusion:**

We found that health-based programs in churches align well with church doctrines, but the success of these programs will depend on, equipping leaders with the skills and knowledge to engage in health-related conversations while including biblically sensitive messaging.

**Supplementary Information:**

The online version contains supplementary material available at 10.1186/s12889-025-23245-7.

## Introduction

There is a high burden of chronic lifestyle-related diseases such as hypertension and diabetes in small island developing states (SIDS) [1–3]. SIDS governments have pursued fiscal policies, public health and healthcare realignment programs and community involvement to decrease the Non-communicable Disease (NCD) burden including the involvement of civil society organizations (CSOs) [4, 5]. Government partnerships with CSOs like service organizations (e.g. Kiwanis and Rotary Club), patient advocacy groups and faith-based organizations have included both health education as well as programmatic intervention. Faith-based organizations (FBOs) have played an important role in the delivery of cardiovascular risk factor reduction programs that have been particularly useful for populations with health disparities [6]. In a recent systematic review, church-based programs were effective in two-thirds of weight reduction and blood pressure control studies and half of physical activity and diet risk factors studies [6].

In the Caribbean, religion continues to be an important cultural reality with approximately 90% identifying as belonging to a religion [7]. Some work has been done to explore feasibility of health programming [8] in churches in the Caribbean but the literature remains limited particularly in understanding the views of church leadership on the implementation and delivery of these programs outside of the United States (US). In the US, church leaders have highlighted the overlap between the role of the church in meeting members’ needs wholistically and the goal of maintaining good health but they have also highlighted challenges in implementation of these programs including insufficient capacity by church leadership [9, 10]. Prior studies have linked FBO-based programming in the Caribbean nations of Barbados, Guyana, Dominica and Jamaica with implementation feasibility and effectiveness, but no specific exploration of church leaders’ views was included [11, 12].

Faith-based organizations have an established history in hosting health promotion programs and have been considered allies in the public health effort to address health matters within at-risk populations [13]. These organizations are led by clergy who are highly esteemed and have tremendous influence over the physical and social environments of their institutions [14]. Understanding church leaders’ perceptions can provide unique insights into the barriers and facilitators that impact the outcomes of health programs within the communities they serve. For example, in a study of Faith-Based Hypertension control for African American communities, church leaders showcased that their engagement resulted in the health program’s acceptance, retention and outcomes [15]. Despite the continued role of religion in the Caribbean, understanding the role of faith-based organizations in promoting health in communities from the FBO perspective is not a well explored area in the region.

Caribbean governments have expressed commitment to adopting and adapting the Chronic Care Model (CCM) [16] to enhance the management of NCDs and many have also adapted and adopted the HEARTS in the Americas initiative [17, 18]. Key components of CCM and HEARTS are community-based health programs which emphasize self-management education [18], and several Caribbean SIDS have begun to implement self-management programs through partnerships with faith-based organizations and other CSOs [19, 20]. Several countries have attempted to implement self-management programs, but we found few reports and no peer-reviewed publications to date on any aspect of the implementation of these programs. Barbados was one of the first countries in the Americas to implement the HEARTS program [21] in response to a high cardiovascular disease burden. This, and similar initiatives target the treatment of hypertension in a clinic-based setting but to date the delivery of community-based components of HEARTS have been limited. Government health officials have expressed commitment to engaging civil society organizations like FBOs to implement these community-centered approaches.

In 2011, Proctor and colleagues described eight implementation outcomes that have promoted conceptual consistency in understanding the process and effectiveness of implementation [22]. From these, Lyons and Bruns later described two types of implementation outcomes– perceptual and behavioural [23]. The former is more suited to the pre-implementation phase of an intervention; understanding them can promote greater chance of implementation success. In a scoping review of ten years of research using the implementation outcomes framework, the authors noted a low proportion of studies in the community and much fewer in the faith-based settings. They called for more studies outside of health care organizations to aid in advancing the theoretical foundations of the framework [24].

In addition to the perceptual outcomes, organizational readiness has been identified as a critical component of successful implementation. Weiner describes organizational readiness as “organizational members change commitment and change efficacy to implement change [25].” Readiness is a multi-level construct and can be difficult to measure and evaluate. The Organizational Readiness for Implementing Change (ORIC) [26] is a 12-item tool which measures organizational members commitment to change as well as their belief that the organization can implement this change.

Given the impetus by local, regional, and international health entities to partner with CSOs to implement self-management and other health education programs, we studied the implementation of a chronic disease self-management program on blood pressure as described in the published protocol. In the overall study we were guided by the Practical, Robust, Implementation and Sustainability (PRISM) framework [27], a component of which highlights the importance of obtaining the organizational perspective. We chose to obtain an organizational perspective from the church leaders’ and in this paper, our primary aim was to explore the three perceptual implementation outcomes which have relevance in the pre-implementation phase- perceived acceptability, appropriateness, and feasibility.

Secondly, we aim to understand perceived barriers and facilitators to implementation as well as organizational readiness from the church leaders’ perspectives with the aim of informing the implementation strategy of the larger study. In this study we use the terms church and faith-based organizations interchangeably and because of the pervasiveness of religion in Caribbean culture we use the church as an example of a civil society organization with which health entities may wish to partner.

## Methods

### Study design and setting

This was a concurrent nested mixed methods study consisting of a quantitative online survey conducted with 52 stakeholders and qualitative individual semi-structured interviews conducted with a subset of 13 stakeholders. The quantitative and qualitative data collection components occurred between February 2021 and March 2022 in 20 churches in Barbados, a country where 80% of the population identify as belonging to a religion [28]. Barbados is classified by the World Bank as a high-income small island developing state where the population of approximately 287,000 consists of 92% Black, 3% White, 3% Mixed and 1.5% Indian descent [28]. Historically, religion has played an important role in the development and socio-political landscape of the island which is home to over 300 faith-based places of worship. Religious clergies are represented in the Senate section of Parliament, on Boards of Management of schools and other major sectors as well as playing active role in the National Non-Communicable Disease Commission. Christianity is the predominant religion, and the island is home to at least 15 Christian denominations.

### Recruitment and data collection

#### Quantitative

In this paper, the term ‘church leader’ refers to a person who leads and manages a single assembly (church) usually ranging in membership from 30 to 200 persons. All these persons held the titles of either reverend, pastor, or priest. The leaders of six Christian denominations and three Christian non-denominational assemblies were approached by the research team (NS, LBG and KL) through letters, email and phone calls made to their administrative offices to invite them and the leaders in their organization to participate in the study. Four denominational leaders agreed to organize virtual meetings for the church pastors/priests serving with them to learn more about the study. Prior to this, churches in Barbados including those invited, delivered ad hoc health fairs, educational talks and exercise programs. The researchers on this study have participated in these activities and so had prior relationships with select church leaders.

During the denominational meetings, church leaders were given a 30-minute presentation on the main concepts and activities found in the chronic disease self-management program (CDSMP) originally developed by Stanford University and administered by the Self-Management Resource Center [29], an independent company created in 2017 to manage the licensing and delivery of CDSMP and similar programs. CDSMP is delivered in six-week workshops aimed at enabling participants to manage their chronic disease(s) effectively [30]. It provides participants with information about seven self-management tools which they can use to help manage their chronic disease: physical activity, medications, decision-making, action planning, breathing techniques and understanding your emotions. The leaders were told that the study targeted those with high blood pressure and this condition, and its complications were explained. The presentation to church leaders included a description of the planned implementation project which would include churches in the implementation of this program. This plan has been described more fully in a previous publication describing the protocol for a trial to be conducted in FBOs in Barbados [31]. Having received the description of the program, a REDCap link was sent for completion of the quantitative survey. The link was shared on the Zoom platform and via WhatsApp to church leaders.

#### Qualitative

For the qualitative interviews, church leaders were purposively selected from the four denominations which replied to our request for corporate presentations with pastors and from non-denominational assemblies who were approached. When choosing leaders, we aimed to have maximal variation in age of the leader and size of the church and all leaders had to have experience implementing a health activity/program in their own church so they could speak from experience on barriers and facilitators. Those sampled for the qualitative study were purposively sampled from among those who were invited to participate in the quantitative survey.

### Data tools and analysis

#### Quantitative

Using the Acceptability of Intervention Measure (AIM), Intervention Appropriateness Measure (IAM) and Feasibility of Intervention Measure (FIM) [32], we assessed acceptability, appropriateness, and feasibility of implementing healthy lifestyle programs in churches. The AIM, IAM, and FIM scales each have four items and in other settings have been tested for and shown to have good content validity, discriminant content validity, reliability, structural validity, structural invariance, known-groups validity, and responsiveness to change [32]. These constructs used together have been found to be particularly relevant to the adoption of an intervention [33]. Additionally, we used a modified version of the Organizational Readiness for Implementing Change (ORIC) (Appendix 1), adapted to the context of the church [26]. For each item on the scales, participants were given the option to respond “1-Completely disagree, 2-Disagree 3- Neither agree nor disagree, 4- Agree 5-Completely agree. We calculated and reported the proportion of persons who responded completely agree/agree with 95% confidence intervals. We calculated mean scores and 95% confidence intervals for each item on the questionnaire and internal consistency (using Cronbach’s alpha) for each measure given that this is their first usage in this population. Analyses were conducted using Stata Statistical Software [34].

#### Qualitative

Thirteen semi-structured interviews were conducted virtually via Zoom with church leaders because of the COVID-19 pandemic. The semi-structured interview guide (Appendix 2) consisted of questions related to the feasibility, acceptability and appropriateness of healthy lifestyle programs in FBOs in general. Interviews were conducted exclusively between participants and interviewers, no one else was included in the sessions. While 14 church leaders were initially invited to be participants in interviews, one female declined to participate. Interviews were conducted by co-authors NS and KN, two female public health researchers, with experience in conducting qualitative research. Sessions lasted 29 min on average. There were no repeat interviews conducted.

Informed consent was obtained by having participants click on a REDCap link which allowed them to read about the study, ask questions and then type their names into REDCap as an expression of consent. The REDCap platform is hosted on a secure server that is HIPAA and GDPR compliant and conforms to the Data Protection Act of Barbados. Sessions were recorded with informed consent and transcribed verbatim. Our interview guide is attached and demonstrates that our questions to pastors included but were not specific to chronic disease self-management programs. We asked questions widely about education and training on healthy lifestyle programs in churches.

Transcripts for church leaders’ semi-structured interviews were analyzed using thematic analysis informed by both inductive and deductive coding. The latter were derived from Proctor’s definitions of the implementation outcomes -acceptability, appropriateness, and feasibility. Using Proctor’s definitions as the basis for our understanding of the three implementation outcomes, we defined our three main constructs as described in Table 1 which provides the basis for our coding framework (Appendix 3) and the way in which the terms were operationalized for this study [22]. In reviewing church leaders’ interviews we considered their perception of their own acceptance as well as their perception of the acceptance of church members.

**Table 1:** Theory-based coding framework

Coding name	Abbreviation	Theoretical Definition	Operational Questions
Acceptability	Acc	The perception that the health programs are agreeable, palatable or satisfactory for the church	Will people accept these programs in the church setting?
Appropriateness	App	Perceived fit, relevance or compatibility of the health program.	How well does the health program fit with the tenets/doctrines of the church?
Feasibility-facilitators	Fea-f	The extent to which health programs can be carried out in the church setting	What are the facilitators to implementing health programs in FBOs?
Feasibility-barriers	Fea-b	The extent to which health programs can be carried out in the church setting	What are the barriers to implementing health programs in FBOs?

All documents were coded by the first author NS using NVIVO 12 software. To ensure validity, codes were reviewed by senior qualitative researcher MM for the first three transcripts and coding framework updated to include the inductively obtained codes and to better explain the definitions being used for feasibility, acceptability and appropriateness. 50% of transcripts were double coded by co-authors KN and LBG and analyzed by the three coders and the senior qualitative researcher. The interviewers and coding team are all public health researchers and members of faith-based organizations who reside in Barbados. MM is a senior qualitative researcher with over 20 years’ experience who guided the development of the semi-structured interview guide, reviewed the interviews and field notes and supervised the analysis. SH is the consultant implementation scientist and mixed methods researcher with significant qualitative research experience. Both interviewers (NS and KN) are trained in qualitative research at the postgraduate level and have over five years’ experience in the area. Interviews were performed until data saturation had been reached. At the eleventh interview we noted comments and themes were being repeated, we continued for two more interviews, including another female to ensure that saturation had truly been reached. While full transcripts were not made available to participants post transcription, participants were sent the parts of the interviews that were included in results for comment and/or correction.

### Integration of quantitative and qualitative methods

A concurrent nested approach was utilized for data analysis with the quantitative and qualitative data first analyzed separately. The quantitative findings are nested within the more dominant qualitative data which was analyzed using thematic analysis. Having coded the data, the core analysis team met during the analysis period to elicit underlying patterns and overarching themes and to determine where and how the rich details emerging from the qualitative data related to the overall thematic areas and gave context to the quantitative findings.

### Ethical considerations

This study was given approval by the University of the West Indies/Ministry of Health and Wellness ethical review board of Barbados (Reference no: 2000504-B-IRB). All participants provided informed consent for their involvement in the study.

## Results

### Description of participants

Of the 107 church leaders to whom the survey was sent, 52 consented and attempted the survey and 40 (39%) responses contained sufficient data for analysis. The median and interquartile ranges for the AIM, IAM and FIM scales were 16 (15-20), 16 (16-20) and 16 (15-17) (out of 20), respectively. On examining individual items, highest percentage of pastors agreed with the item- “Chronic Disease Self-management program in church seems possible” (97%) and the lowest percentage was found for the item where leaders responded to the statement: “Chronic Disease Self-management program in church seems easy to use” (60%). Given that this was the first use of the AIM, IAM and FIM questionnaires in this population, we assessed the internal consistency of the scales using Cronbach’s alpha and the scores were as follows: 80% - AIM; 96% - IAM; 82% - FIM.

For the semi-structured interviews, we conducted thirteen interviews with church leaders from four Christian denominations- New Testament Church of God, Wesleyan, Anglican, Nazarene, and an Independent Assembly. There were nine males, and four females interviewed, ranging in age groups from 35 to 75 years old (Table 2).

**Table 2:** Participants of semi-structured interviews

Code	Gender	Age group
PM1	Male	30–40
PM2	Male	30–40
PF3	Female	60–70
PF4	Female	70s
PF5	Female	50–60
PM6	Male	50–60
PM7	Male	70s
PM8	Male	40s
PF9	Female	60–70
PM10	Male	40–50
PM11	Male	40–50
PM12	Male	60–70
PM13	Male	40–50

### Acceptability

The quantitative survey showed high levels of acceptability, between 77 and 92% of respondents indicating that the self-management program as described to them would be acceptable within the church (Table 3). This assessment was in alignment with the overall findings from the semi-structured interviews. Church leaders generally indicated that it would be acceptable to them but held mixed views around how acceptable the program might be to their members.

**Table 3:** Mean and proportional scores for each item on the acceptability of intervention measure scale

Survey items	Mean (95% CI)	Percentage who agree/strongly agree%, (95% CI)
Acceptability of Intervention Measure		
Chronic Disease Self-management program in church meets my approval.	4.0 (3.7,4.3)	82 (69, 95)
Chronic Disease Self-management program in church is appealing to me.	3.9 (3.6, 4.3)	77 (63, 91)
I like Chronic Disease Self-management program in church.	4.0 (3.7, 4.3)	82 (69, 95)
I welcome Chronic Disease Self-management program in church.	4.3 (4.0, 4.5)	92 (83, 100)

### Acceptability: church leaders describe palatability of health programs

Mirroring the quantitative findings, church leaders were very positive about acceptability of self-management programs within the church. Church leaders consistently referred to the role the church needed to play in promoting healthy lifestyles.

Some leaders highlighted their acceptance of the integration of healthy lifestyle programs within the church by providing examples of those that already existed:

*We have many presentations for health. We look at different areas of the Women’s Health*,* men’s health and the total man. We had an exercise program but because of COVID that has stopped a little bit* (PF5,).

*We had a chef who is one of our members and he started teaching about healthy living and healthy cooking… how to do your chicken and so on without frying and trying to do your vegetables and you know steaming and so on* (PF3).

Many leaders also saw the church as having a health promotion role through making education/advice and diagnostic checks more readily accessible to members:

*Some persons have an aversion to going to a doctor um…or don’t have a lot of patience going to the clinic*,* um… Whatever the reason might be so here’s an opportunity to come and get some of those things checked without having to pay any money without any hassle whatever the care maybe and then you get back the results and then you can you know be given guidance as to what to do from there* (PM13,).

### Acceptability: pastors perception of members’ acceptability of health programs

While church leaders were confident in their own acceptance of health programs, they held mixed views on the likely acceptability to members. Only 63% (95% CI: 48%, 79%) felt that members were determined to implement healthy lifestyle changes in their own lives and 63% felt that their members feel confident that they can keep track of progress made in creating a supportive healthy environment. During interviews, leaders revealed that in their attempts to change the content of their sermons to include more health-related content, they may experience some “backlash”.

*]So like half*,* not half*,* like about a third of the service*,* every Sunday is going to be about health… So I know*,* um how some persons may say to me that they prefer to have the entire service on about God and Jesus…And less doing with seems spiritual*,* so I know I may get some backlash there in terms of that*,* but I know it may be a challenge* (PM1).

While church members welcomed health education and screening activities there was resistance to programs that tried to introduce individual level care as some saw this as a replacement of their personal health care provider.

*Because in their mind this is a medical situation. I already trust this particular doctor. I don’t need to back talk to the pastor about this. I will go to the doctor. We find that mainly that is what would happen* (PM13).

Finally, as it relates to perceived members’ acceptance, church leaders felt that even those members who might be amenable to such a program might have difficulty in maintaining healthy lifestyle recommendations. These acceptability related barriers may affect the feasibility of implementation.

### Acceptability: facilitators perceived to enhance acceptability

Church leaders provided ideas on what actions could facilitate implementation of health programs in churches. Two themes emerged around facilitating acceptability of the programs: building and maintaining trust and simplifying materials and educational resources.

1. Build and maintain trust: There was a sense that people were generally skeptical of some health information and of taking medications. Leaders therefore suggest that before health information is shared, collected and used, researchers and health educators should work on building the trust of congregants.

*So I think um… the challenges is to gain people’s confidences that you are not going to be um… the information you get will be used confidentially.* (PM7).

2. Keep materials and questionnaires simple and avoid jargon: This was an important theme for the church leaders who felt that some of the information presented in past programs was not at a literacy level that members and the community could understand.

*Where possible you have a simple questionnaire and probably make it easier…for people to respond. That’s why I said simplicity in the questionnaire. So that’s another thing*,* the language that… is used… I know you can’t really adulterate the terms but if it can be simplified it would help* (PM7).

*“So in other words being able to use less jargon but not water down the facts of what you are sharing.”* (PM13).

### Appropriateness: healthy lifestyle programs are aligned with doctrine

The levels of appropriateness were high in both quantitative and qualitative assessments. Approximately 90% of church leaders believed these programs are appropriate (Table 4). In the interviews, they explained that health was a priority for the church as it deals with the whole man. Interestingly, some were keen to highlight that the church’s priority is spiritual health and thus that must take precedence over all else.

**Table 4:** Mean and proportional scores for each item on the intervention appropriateness measure

Survey items	Mean (95% CI)	Percentage who agree/strongly agree%, (95% CI)
Chronic Disease Self-management program in church seems fitting.	4.3 (4.0, 4.6)	90 (80, 100)
Chronic Disease Self-management program in church seems suitable.	4.2 (3.9, 4.5)	88 (77, 98)
Chronic Disease Self-management program in church seems applicable.	4.3 (4.0, 4.6)	90 (80, 100)
Chronic Disease Self-management program in church seems like a good match.	4.3 (4.0, 4.5)	90 (80, 100)

Church leaders consistently aligned healthy lifestyle programs with the tenets/doctrines of their faith irrespective of denomination. Leaders frequently quoted from various passages of scripture. Most commonly, pastors mentioned the concept that the body is the “temple” of God which should be treated with care. Some leaders quoted from the Marks of Mission which express the Anglican community’s common commitment to, and understanding of, God’s holistic and integral mission and used doctrinal commitments from other denominations to demonstrate alignment.

*Well the churches role should be to promote the healthy eating and physical activity um from a biblical standpoint*,* if I may use that because the body is the temple and we have been commissioned to take care of the temple* (PM2).

*The body was created by God. It’s good. and part of our responsibility as the Psalmist tells us he made us a little lower than the angels to take care of God’s created order and part of what was created is the body. from managing illnesses*,* managing ourselves is part of that role.* (PM11).

### Appropriateness: health programs are appropriate but spiritual health is the priority

Similar to what was described when addressing the acceptability of chronic disease self-management programs in churches, the issue of spirituality being seen as the priority of the church was discussed:

*The primary health concern is the spiritual health*,* of course we must also be concerned with our physical health*,* our mental health*,* or emotional health…. The major challenge that I see… when we would do it. Do we sacrifice….…like our bible study time or pray time*, (PM13)

### Appropriateness: facilitators perceived to enhance appropriateness

Church leaders provided ideas on approaches in health programming that may enhance the appropriateness of health programs in churches. They felt that using a biblical perspective, offering practical solutions and creating family-oriented programs would increase the likelihood that health programs are more compatible and relevant in the church setting.

1. Use a Biblical perspective: Our respondents felt that using biblical verses and examples would enhance the adoption of the program within churches and increase its success.

*And I find when you are dealing with um Christians*,* you have to bring a biblical perspective. Why they should not do this and shouldn’t do that and*,* you understand?………. Especially when you are dealing with the older folks* (PF9).

2. Healthy programs in churches should offer practical solutions: Church leaders felt that health care providers focused too heavily on outlining the health problems without clearly defining solutions.

*Just you know practical ways in which the particular cause I know sometimes from sessions not at our church but I’ve been to sessions before that people literally left depressed cause it was like okay so I am dead. There is nothing. I just heard about a million things that are probably wrong with me and…there was no solution given right?* (PM13).

3. Create family programs: There was the recognition by some of our leaders that health conditions and outcomes should be addressed across the life course. Thus, they called for an approach to healthy lifestyle programs that considers integration of the whole family.

*Put a spin on it so that it is family oriented*,*……. So get the whole family involved. Because these matters…these issues. I do believe there*,* there are some children some teenagers*,* who are afflicted with these illnesses as well* (PM11).

*We’ve done a lot on families as well. Because strong families for me builds strong communities and strong churches* (PF3).

### Appropriateness: the relationships between food, culture, and church fundraising events

As is culturally typical in Barbados and the wider Caribbean, big events such as church fundraisers are food-centered events. Many foods high in salt, fat and sugar are also culturally accepted foods that are expected to be sold at these events to attract patrons. Anecdotally, the relationship between these foods (e.g. coconut sweetened bread, macaroni pie-a cheese-filled pasta dish, and fish cakes) and fundraising is strong; healthy items have less selling power. Yet having such items at church events would undermine chronic disease self-management programs.

*At church events we sell a variety of things……we do healthy foods we have those people who would promote healthy foods*,* but you know the Barbadians still like them fishcake (fried fish and flour)… and them souse (pickled pork) and those kind of things so we cater to them. We have fruit*,* we have vegetables we have fruit drinks*,* we prefer not to use the… bottle drinks and you know the drinks like the sodas* (PF4).

There is a fear that removing unhealthy items for sale would challenge the financial success of such events and the church financial bottom line. This fear of financial threat also affects the perceived feasibility of healthy programs.

### Feasibility: pandemic effects on health programs in churches

For three of the four quantitative items, more than 85% of church leaders indicated that chronic disease self-management programs in churches seem implementable, possible and doable (Table 5). Only 60% thought that such a program would be easy to use/follow. In semi-structured interviews, church leaders shared their ideas on the feasibility of the programs in churches by drawing on experiences they had had previously with health programs in churches. Based on these experiences, they were able to share potential barriers and provide advice on possible facilitators. They spoke about the pursuit of health and wellness activities such as hiking and health fairs; however, the Covid-19 pandemic hindered many of these "health-related activities” creating a barrier to positive health practices such as physical activity and social interaction.

**Table 5:** Mean and proportional scores for each item on the feasibility intervention measure

Survey items	Mean (95% CI)	Percentage who agree/strongly agree%, (95% CI)
Chronic Disease Self-management program in church seems implementable.	4.1 (3.9, 4.3)	88 (77, 98)
Chronic Disease Self-management program in church seems possible.	4.3 (4.1,4.5)	97 (92, 100)
Chronic Disease Self-management program in church seems doable.	4.2 (4.0, 4.4)	92 (84, 100)
Chronic Disease Self-management program in church seems easy to use.	3.6 (3.4, 3.9)	60 (44, 76)

Some church leaders felt that the economic impact of the pandemic, specifically the loss of income due to COVID-19 contributed to poor dietary habits in people, because of the lack of affordability of nutritious foods which are considered to be more expensive.

*Due to COVID etc.*,* a lot of persons are no longer working. Finances have become a problem*,* and healthy eating is expensive unfortunately* (PM2).

This COVID-19 impact is likely to continue beyond the pandemic for both the individual and the church as an organization.

### Feasibility: church leaders not equipped to deliver health-related topics

Despite acknowledging the appropriateness of health programming within the church, some leaders expressed that they felt ill equipped to address health-related topics themselves.

*I know how to preach the word but I don’t know how to ask people about how they are feeling regards with regards to disease and diabetes and hypertension so putting someone in place like that to be able to communicate with them is important*, (PM8).

Many indicated the need for a focal point/health champion within the church with the knowledge on how to communicate these issues appropriately, to assist in this regard.

### Feasibility: human resource barriers and facilitators

Church leaders felt that individual and committee champions needed to be assigned to the health programs to enhance the likelihood of success They felt that health champions should be identified from among church members but anticipated that they may experience challenges in finding persons interested in taking up this role.

*We would have to put some person in charge of it*,* yes*,* we have church clerk and we have you know the normal church structure*,*……you would have to put somebody in charge of it to be responsible for it and make sure that everything is recorded and given to the Church. Cause to go and put this on um on the system now*,* it will be too much* (PM11,).

*Finding the person might be the hardest part now to want to take up that role.”* (PM10).

### Feasibility: churches’ experience in community programs including health

Church leaders described their community’s experience in engaging community fairs which incorporated elements of health promotion. The successful implementation of these events is an indicator that more consistent health programming may be both feasible and acceptable.

*As part of our evangelistic effort um… every summer we had a major outreach in the (Community name) and we would setup tents and up jumping tents you know all these things and we would have a tent where you would get the same um… health checks being done*,* blood pressure checks*,* checking of heart rate*,* these different things happening there. So in that instance that would be an annual exercise* (PM13).

In addition to community fairs, leaders mentioned other health-related community activities that included the creation of kitchen gardens with children, exercise/gym activities on the church premises and line dancing.

### FBO readiness to reach communities

Using the modified ORIC survey, we asked pastors about their perceptions of the church’s readiness to be a community hub for healthy lifestyle programs. The majority of church leaders (90%, 95% CI 81, 99) felt that people who attend their church wanted to promote healthy living and 83% (95% CI, 72%, 95%) felt confident that the church could serve as a community hub for healthy lifestyle programs; From our inductive coding, a strong theme emerged around the impetus of the church organization to reach out to their neighboring communities for special projects and events. In alignment with the findings from the readiness survey, church leaders freely discussed their activities in the communities explaining the extent to which the church could be used not simply as a mode to improve members’ health but as a vehicle by which the wider community around the church can be reached. Leaders viewed community involvement and outreach as part of their mission as churches and described it as aspects of their “compassionate” ministry.

*Well sometimes when we have activities we don’t only restrict it church members. We would have suppose… there is a seminar we would encourage persons to invite their family and friends um… who are obviously not members of the church as well*,* so in a way you are helping them in reaching out in evangelism but at the same time we also reacting to a need that they would have so that…cause I don’t think as I said that the church is there just to preach at people…* (PM7).

Churches have engaged their neighboring communities using both individual level recruitment and household focused invitations, knocking on doors of houses within the communities to mass media communication using flyers placed in mini-marts and local gas stations and social media platforms like WhatsApp, Facebook, and Instagram. The use of virtual platforms has increased access to those who did not previously attend. On the virtual platforms, church leaders have noticed more community/ non-member attendees.

### Readiness: churches need health professional support to deliver programs

Even though church leaders believe that churches can create a facilitative environment for behavior change through supporting of health programs within the church delivering health messages from the pulpit, some did not feel equipped enough to deliver the messages themselves. They described that they have invited health professionals to deliver these messages and indicated ways in which the professionals can improve their delivery and the appeal of the health message. While they have used professionals of different faiths/beliefs, their preference was for persons of the same faith who understood the culture of the faith. One pastor indicated that he prefers to use the doctors from his own congregation because they “know the people” and the congregation “has confidence in them”- indicating the value of relationship, trust and the power of knowing the culture of specific faith.

## Discussion

We found that health-based programs in churches have a high chance of being successful because of doctrinal alignment with church tenets but that the success of these programs will depend on establishing trust through engagement of church-based champions, tailoring programming to include a biblical perspective, and engaging the entire family. Success of programs will also require strengthening health-related capacity of FBO and providing necessary support to deliver health-related programming,

Our work is novel in its use of implementation tools (AIM, IAM and FIM) in assessing likelihood of health program adoption to a Caribbean FBO community in the pre-implementation phase and demonstrates high reliability of these instruments. Both our quantitative and qualitative findings suggest that health programs are perceived as appropriately aligned with the doctrines and mandate of the church. Church leaders expressed their acceptance of healthy lifestyle programs in churches but held mixed views on the perceived acceptance of their congregants/members. This perceived alignment of health and church programs has been mirrored in church studies in African American and Latino churches across the United States [14, 15, 35].

Church leaders are believed to have significant influence over their members and are thus poised to influence health behavior [13, 28, 36, 37]. Therefore, some authors have recommended that one of the key elements for community-based health programs identified is commitment from church leaders to provide health education to the congregation [38, 39]. In our study, the church leaders felt that they would need considerable support from health care providers to adequately deliver sustainable health promoting programs in churches. They specifically expressed a preference for health care providers who understand and are respectful of their church culture. This signals the need for closer collaboration between the church and health system in this setting.

### Implications for health program implementation in FBOs

Church fundraising is a potential source of unhealthy food availability for congregants and the community. Attempts to introduce health programs need to be sensitive to this challenge. Alternatively, fundraising could be centered around activities other than food to reduce the temptation of having high salt, high fat, high sugar, items. In our study, churches reported having health walks and it may be advisable for them to also include hikes, line dancing and other modalities that emphasize health. These latter activities have become increasingly common in Barbados in alignment with initiatives by the National Taskforce on Wellness [40]. These church leaders reported significant activity within the community which can be leveraged to promote health programs while strengthening evangelistic outreach for the church.

This study was conducted during the COVID-19 pandemic thus the pandemic public health measures had a significant impact on the church leaders’ perception of barriers to delivery. The economic factors which leaders felt might be barriers to healthy eating even prior to pandemic were exacerbated during this time. Other studies highlighted the financial challenges members experienced in attempting to live healthier [41]. During the pandemic, the World Bank reported that “the crisis had a dramatic impact on global poverty and inequality” [42]. Studies in Thailand and Philippines found that COVID-19 pandemic impacted the affordability of healthy diets particularly for the poor through a reduction in their incomes [43]. These global perspectives are reflected in the observations of church leaders in this study.

### Strengths and limitations

Our study used robust measures and constructs of acceptability which has not always been the case in the literature and is rare in Caribbean implementation studies. Acceptability, appropriateness and feasibility have together been used to predict adoption and our use of Proctor’s implementation outcomes in a mixed methods study is not common in low -middle income or small island developing countries. Faith-based organization membership has a high proportion of women and thus our learnings from this setting may reflect that inherent organizational bias. We did, however, interview more men than women since there are more male leaders in the denominations sampled than female leaders.

While having the male leadership perspective is valuable, it may also limit our understanding of the barriers and facilitators and the readiness of the organization since FBOs have a high proportion of women and the male leaders may present a male dominated understanding of members readiness for healthy lifestyle programs.

To expand the reach of healthy lifestyle programs and promote equitable delivery, partnerships must be wider and include sports, patient led and other community-based organizations. We had a low response rate in the quantitative survey and those who responded may have been more likely to have an interest in healthy programs and thus gave what they perceived to be favorable responses.

This study was done while the country was also handling the consequences of COVID-19 pandemic during periods characterized by lockdowns. Leaders may have been unfamiliar with the online platform used for the meetings and the questionnaire links thus limiting their response. People unfamiliar with technology are more likely to be older and had they participated their responses may have been different from those who did. The low response rate limits the representativeness and transferability of the findings.

Some of the information from this study may be transferable to other CSO settings but further work in one or more of these settings is needed to understand the differences and to correct the gender bias in recruitment. This study was limited to the church leaders’ perceptions and did not examine the views of members on appropriateness, acceptability, and feasibility of health programs in the church organization.

## Conclusion

Given the World Health Organization’s endorsement of the integration of FBO communities in achieving the Sustainable Development Goals, studies such as this one can assist other Caribbean SIDS in understanding transferable lessons learnt in health program implementation. We found high acceptability and appropriateness for health programming in churches. However, program planners and policy makers should note church leaders reported discomfort with addressing health-related topics. The success of these programs will depend on equipping leaders and members, establishing trust through the engagement of church-based champions and tailoring programs to include biblically sensitive messaging.

## Electronic supplementary material

Below is the link to the electronic supplementary material.

Supplementary Material 1:: Appendix 1: Modified Organizational Readiness for Implementing Change (ORIC).Appendix 2- Semi-structured Interview Guide. Appendix 3- Codes as used in NVIVO 12

## Data Availability

The datasets generated and analysed during the current study are not publicly available due to the relatively high probability of information being identifiable given the small size of the island population. The data are available from the corresponding author on reasonable request.

## Abbreviations

AIM: Acceptability of Intervention Measure
CCM: Chronic Care Model
CDSMP: Chronic Disease Self-Management Program
CSOs: Civil Society Organizations
FIM: Feasibility of Intervention Measure
FBO: Faith-based organization
IAM: Intervention Appropriateness Measure
NCD: Non-communicable Diseases
SIDS: Small Island Developing States
