# Supplementary material for: Faith-based leaders’ perceptions on the implementation of programs to promote healthy lifestyles in churches in Barbados- a mixed-methods analysis
Source: BMC Public Health. 2025 Jul 2;25:2191. doi: 10.1186/s12889-025-23245-7 (PMC12220731; doi:10.1186/s12889-025-23245-7)
Supplement: Supplementary file 1 — Supplementary Material 1:: Appendix 1: Modified Organizational Readiness for Implementing Change (ORIC).Appendix 2- Semi-structured Interview Guide. Appendix 3- Codes as used in NVIVO 12 [file 12889_2025_23245_MOESM1_ESM.docx]

Appendix Files

**Appendix 1: Modified Organizational Readiness for Implementing Change (ORIC)**

| 1 | 2 | 3 | 4 | 5 |
| --- | --- | --- | --- | --- |
| Disagree | Somewhat  Disagree | Neither Agree nor Disagree | Somewhat  Agree | Agree |

| 1. People who attend church here feel confident that the church can be a community hub that encourages healthy lifestyle changes. | 1 2 3 4 5 |
| --- | --- |
| 1. People who attend church here are committed to creating healthy lifestyles | 1 2 3 4 5 |
| 1. People who attend church here feel confident that they can keep track of progress made in creating a supportive healthy environment. | 1 2 3 4 5 |
| 1. People who attend church here will do whatever it takes to promote and support healthy lifestyles. | 1 2 3 4 5 |
| 1. People who attend church here feel confident that the organization can support people as they adjust healthier living. | 1 2 3 4 5 |
| 1. People who attend church here want to promote healthy living. | 1 2 3 4 5 |
| 1. People who attend church here feel confident that they can keep the momentum going in promoting healthy lifestyle changes. | 1 2 3 4 5 |
| 1. People who attend church here feel confident that they can handle the challenges that might arise in promoting and supporting healthier choices. | 1 2 3 4 5 |
| 1. People who attend church here are determined to implement healthy lifestyle changes. | 1 2 3 4 5 |
| 1. People who attend church here feel confident that they can coordinate tasks so that implementation of healthy events and activities goes smoothly. | 1 2 3 4 5 |
| 1. People who attend church here are motivated to implement this change. | 1 2 3 4 5 |
| 1. People who attend church here feel confident that they can manage the politics of changing to healthier diets and more physical activity. | 1 2 3 4 5 |

**Appendix 2- Semi-structured Interview Guide**

General role of church in health

1. What do you think is the role of the church is in supporting healthy lifestyles

*Prompt: physical activity and healthy eating)?*

2. Tell me a little about what your church is doing to support good health for members

*Prompt: eg. talks and demonstrations by nutritionists?*

Healthy lifestyle programs

2. Tell me about what your church is doing to support healthy lifestyles for members

*Prompt: do you have health walks? An exercise program like line dancing? eg. do you have nutrition classes? Fruits and vegetables/Sweets sold at church events?*

3. What is the role of medication in controlling chronic diseases?

Community involvement

4. How do your activities target the wider community? (Allow answer)

*Do your health programs target the church or do they also include the community?*

*Follow up: if so, how do they target the wider community?*

5. What methods does the church use to reach out to the community?

Challenges to healthy lifestyle programs

6. How do you think members would feel about a structured lifestyle program that involves workshops for six weeks?

7.What are some of the challenges you foresee in the church promoting healthier lifestyles?

*Follow up: How might you/the church help people overcome these challenges?*

*If leader lists then go through each one.*

8. How can health professionals (nurses, doctors, nutritionists) help you promote healthy lifestyles?

*Prompt: Are there health professionals within the church or outside the church who have helped? How have they helped?*

Monitoring and evaluation

9. What are the responses from your church members on the healthy lifestyle programs/activities?

10. How do you obtain feedback from the members on how well the programs are working?

**Appendix 3- Codes as used in NVIVO 12**

|  |  |  |
| --- | --- | --- |
|  |  |  |
| Name | Files used | References |
| Acceptability | 1 | 1 |
| To participants of workshops | 1 | 27 |
| To the leader | 13 | 58 |
| To the people as perceived by the leader | 10 | 26 |
| To workshop leaders | 1 | 17 |
| Appropriateness | 14 | 42 |
| Church's role | 6 | 17 |
| Health | 3 | 3 |
| Community activities | 2 | 3 |
| Narrow church commuity | 13 | 44 |
| Wider neighbourhood community(outreach) | 14 | 63 |
| Feasbility-facilitators | 13 | 44 |
| workshop | 1 | 33 |
| feasibility-barriers | 14 | 65 |
| workshops | 1 | 9 |
| Foods- perceived as healthy and unhealthy | 2 | 6 |
| Links to health professionals | 0 | 0 |
| Inside the church | 7 | 18 |
| Outside the church | 10 | 20 |
| Perception of health | 3 | 3 |
